# Cardiac Tissues From Stem Cells: New Routes to Maturation and Cardiac Regeneration

**DOI:** 10.1161/CIRCRESAHA.121.318183

**Published:** 2021-03-19

**Authors:** Giulia Campostrini, Laura M. Windt, Berend J. van Meer, Milena Bellin, Christine L. Mummery

**Affiliations:** 1 Department of Anatomy and Embryology, Leiden University Medical Center, Leiden, the Netherlands (G.C., L.M.W., B.J.v.M., M.B., C.L.M.).; 2 MESA+ Institute (B.J.v.M.), University of Twente, Enschede, the Netherlands.; 3 Department of Applied Stem Cell Technologies (C.L.M.), University of Twente, Enschede, the Netherlands.; 4 Department of Biology, University of Padua, Italy (M.B.).; 5 Veneto Institute of Molecular Medicine, Padua, Padua, Italy (M.B.).

**Keywords:** cardiac tissue, drug discovery, heart failure, humans, stem cells

## Abstract

The ability of human pluripotent stem cells to form all cells of the body has provided many opportunities to study disease and produce cells that can be used for therapy in regenerative medicine. Even though beating cardiomyocytes were among the first cell types to be differentiated from human pluripotent stem cell, cardiac applications have advanced more slowly than those, for example, for the brain, eye, and pancreas. This is, in part, because simple 2-dimensional human pluripotent stem cell cardiomyocyte cultures appear to need crucial functional cues normally present in the 3-dimensional heart structure. Recent tissue engineering approaches combined with new insights into the dialogue between noncardiomyocytes and cardiomyocytes have addressed and provided solutions to issues such as cardiomyocyte immaturity and inability to recapitulate adult heart values for features like contraction force, electrophysiology, or metabolism. Three-dimensional bioengineered heart tissues are thus poised to contribute significantly to disease modeling, drug discovery, and safety pharmacology, as well as provide new modalities for heart repair. Here, we review the current status of 3-dimensional engineered heart tissues.

In 1998, James Thomson described the derivation of pluripotent human embryonic stem cells (hESC). It was only a matter of time before beating cardiomyocytes were identified in differentiating cultures and inevitable that human induced pluripotent stem cells (hiPSCs), derived around 10 years later,^1^ also differentiated to beating cardiomyocytes. Studies using healthy or patient-derived hiPSC cardiomyocytes for transplantation to repair the heart after damage and in vitro disease modeling were rapidly initiated. Channelopathies caused by mutations in cardiac ion channels were most abundantly studied^2^ and cell transplantation advanced,^3^ but immaturity of hiPSC cardiomyocytes has remained an obstacle: they fall short in mimicking the biological and physiological phenotype of cardiomyocytes in the 3-dimensional (3D) heart in vivo. Recent evidence shows that cardiac tissue engineering may address this and thus accelerate use of hiPSC in preclinical drug testing, identifying mechanisms underlying genetic and acquired human heart disease and in cardiac regeneration therapy. Here, we present the latest engineered models based on cardiac derivatives of human pluripotent stem cells (hPSCs) and discuss their applications in human heart research.

## Why Do We Need 3D Cardiac Models?

Models to study any question are ideally as simple as possible and as complex as necessary. In that sense, there would be no need to increase the complexity of current heart models if simpler models—2-dimensional (2D) cardiomyocytes or noncardiomyocytes expressing cardiac genes ectopically—reflected physiology in vivo or predicted drug responses accurately. Simple models are often easier to implement than their 3D counterparts. For example, single-cell patch-clamp electrophysiology in substrate-attached cardiomyocytes is still the gold standard to quantify parameters such as upstroke velocity, membrane potential, and ion currents. Similarly, voltage-sensitive dyes and multielectrode arrays were developed for use in 2D monolayer cultures. These simple models have been widely used to detect disease and drug effects in hPSC cardiomyocytes and even though highly reductionist versions of the real heart, they often reproduce responses of native tissue. Examples include ion channel mutations causing long QT syndrome (eg, mutations in *KCNQ1*),^4^ Brugada syndrome (mutations in *SCN5A*),^5^ and catecholaminergic polymorphic ventricular tachycardia (mutations in *RYR2*).^6^ In addition, drug-induced changes in electrophysiology^7–10^ or contractility^11–13^ or combinations of both^14^ in 2D hPSC cardiomyocytes have been shown to predict toxic effects on the heart accurately, in some cases better than standard animal models.

Not uncommonly, however, single-cell or 2D models fail to capture expected phenotypes. Typically, this is the case for genetic defects that impair structural proteins, such as myosin-binding protein C or desmosomes, associated with hypertrophic cardiomyopathy and arrhythmogenic cardiomyopathy (ACM), respectively. While phenotypes have been revealed in hPSC cardiomyocytes cultured in media that promote maturation in single-cell and 2D formats in the case of hypertrophic cardiomyopathy,^15^ ACM required not only structural and metabolic maturation but also stress conditions, complex 3D models,^16–19^ and noncardiomyocyte populations.^20^ Similarly, for mutations in *TTN*, the gene encoding the large sarcomere protein titin causing dilated cardiomyopathy, contractility in hiPSC cardiomyocytes was unchanged in 2D while it was impaired in 3D. Enhanced hPSC cardiomyocyte maturation in 3D may be one reason for this, besides more physiological load on the cardiomyocytes. Load directly changes the output force and work that cardiomyocytes need to deliver^21^; this might enhance maturation. For drug-induced changes in electrophysiology or contractility, immaturity might explain why certain drug classes such as PDE3 (phosphodiesterase enzyme 3) inhibitors do not affect single-cell or 2D hPSC cardiomyocytes (which do not express PDE3A) while 3D hPSC cardiomyocytes (expressing PDE3^20^) respond as expected.^22^

While considerable effort has been made to develop highly efficient differentiation protocols and enrich for hPSC cardiomyocytes in differentiating hPSC cultures, noncardiomyocytes increase both contractility and electrophysiology, so that in recent studies, cardiomyocytes are often no longer purified or noncardiomyocytes are added before creating 3D tissues. Coculture conditions are still being optimized: for example, addition of cardiac fibroblasts (CFs) promotes cellular organization, but high CF:cardiomyocyte ratios in a cardiac tissue can cause conduction blocks, increase stiffness, and slow down conduction.^23^ Correct substrate stiffness^24^ and medium components^25^ are essential for physiologically relevant cardiac responses.

For more complex cardiac disease, it may be important to include inflammation and reduce oxygen (hypoxia) as in myocardial infarction (MI) or mimic fibrosis that follows heart damage. Adding inflammatory components (eg, macrophages and inflammatory proteins) or vascularization requires more advanced 3D models. These are models in which cells can grow or move in the 3D space. In the following sections, we discuss what they have contributed to the field so far.

## 3D Cardiac Models

### Scaffold-Free Cardiac Tissues

**Figure 1.**
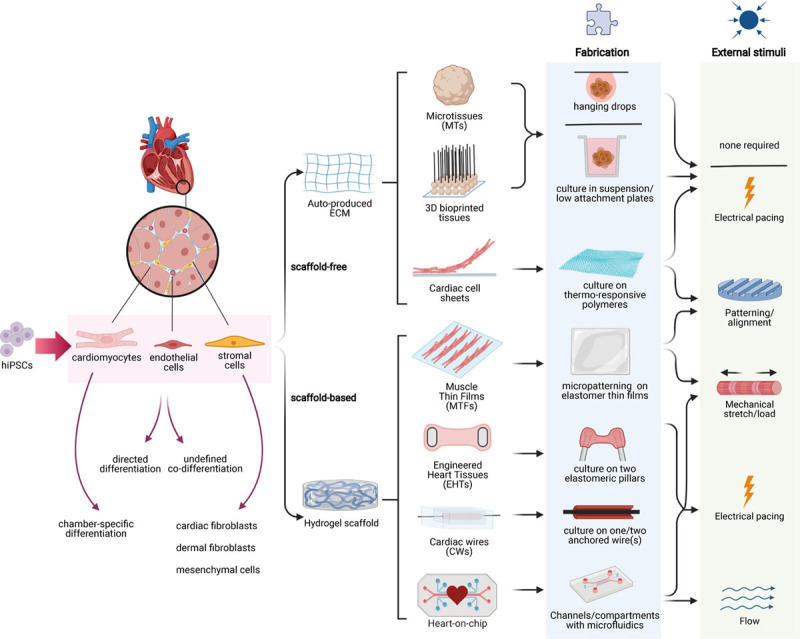
**Three-dimensional (3D) cardiac models.** Overview of scaffold-free and scaffold-based 3D cardiac models. **Left**, The main cardiac cell types (cardiomyocytes, endothelial cells, and stromal cells) included in multicell type 3D cardiac models and can be derived either from primary (animal) tissue or from human induced pluripotent stem cells (hiPSCs). **Right**, Fabrication methods and external stimuli for each type of cardiac model are shown schematically. CW indicates cardiac wire; ECM, extracellular matrix; EHT, engineered heart tissue; MT, microtissue; and MTF, muscle thin film.

Scaffold-free cardiac tissues, in whatever form or combination of cell types, are self-organizing structures in which there is cross-talk between cells; they position within structures and deposit their own ECM (extracellular matrix). They fall broadly into 3 categories (Figure 1): cardiac microtissues, 3D-bioprinted tissues, and cardiac cell sheets. A hybrid variant, where a mixture of hiPSC cardiomyocytes and fibroblasts anchored to the substrate in a dog bone shape that allows uniaxial stress on the 3D tissue, has also been reported.^26^

#### 3D Cardiac Microtissues

Cardiac microtissues are spheroids of heart cells usually formed by self-aggregation. The process is rapid and generally does not require specific equipment. Direct cell-cell contacts and paracrine cell communication take place within the spheroids, followed by ECM deposition and interaction in the tissue microenvironment, much as in normal heart development. Cardiac microtissues from hPSCs are similar to hPSCs differentiated in aggregates called embryoid bodies, except that the cardiac input cells are predifferentiated, whereas in embryoid bodies, differentiation is directed by growth factor addition or is spontaneous, so that derivatives of germ layers other than mesoderm may be present.^27^ Cardiac microtissues can be formed from small cell numbers and are usually <300 μm in diameter so that oxygen and nutrients reach the center of the tissue.^28,29^ Aggregation itself is facilitated by suspending cells in hanging drops,^30–32^ in rotating suspension cultures for 2 days,^33,34^ culture in nonadhesive agarose hydrogel molds,^35^ or most commonly in low-attachment plates.^11,20,36–40^ Early studies using primary neonatal rat cardiomyocytes showed self-aggregation in 3D, spontaneous and synchronous beating, ECM production, and retention of cardiomyocyte properties.^41,42^ Addition of endothelial cells (ECs) to these microtissues resulted in vascular-like structures forming and inclusion of fibroblasts improved cell viability, self-organization, and contractile function.^36,43^ Similar results were obtained using hPSC cardiomyocytes in microtissues containing ECs or fibroblasts, or both. Input ratios of the different cell types in microtissues can be controlled and optimized to recapitulate the cellular composition of the human fetal or adult heart (either in cell number or occupied volume). Ratios reflecting those in the fetal heart, where there are twice as many cardiomyocytes to noncardiomyocytes than in adult heart, enhanced microtissue contraction, structure, and function^35^ although other studies using different ratios showed similar effects (Tables 1 and 2).

**Table 1.**
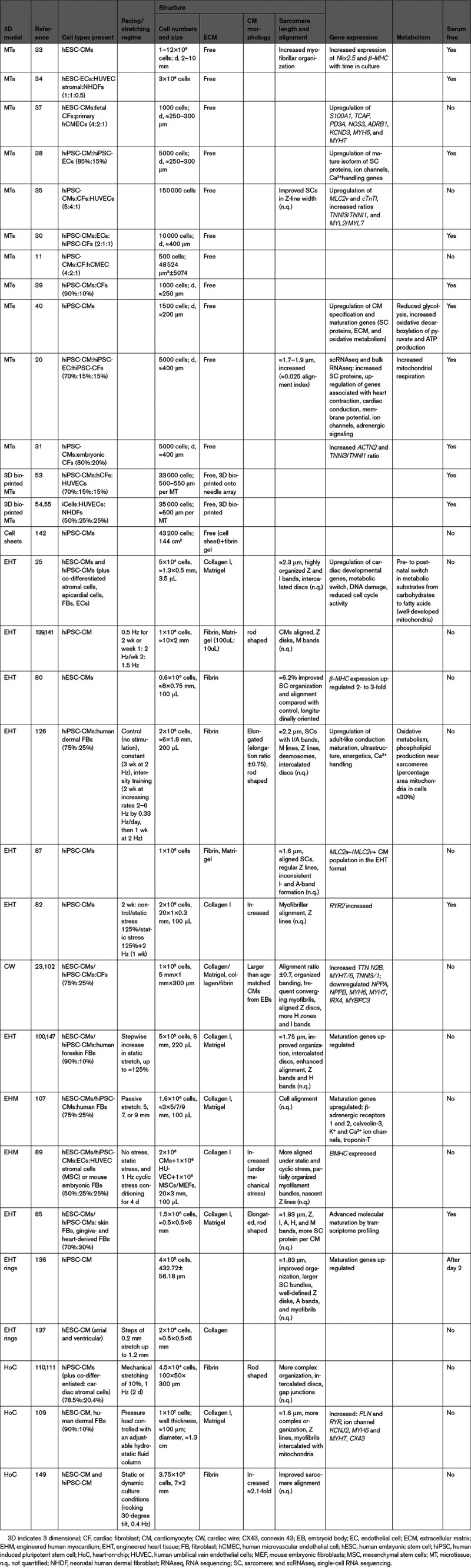
Composition, Structure, Gene Expression, and Metabolism of 3D Cardiac Tissues

**Table 2.**
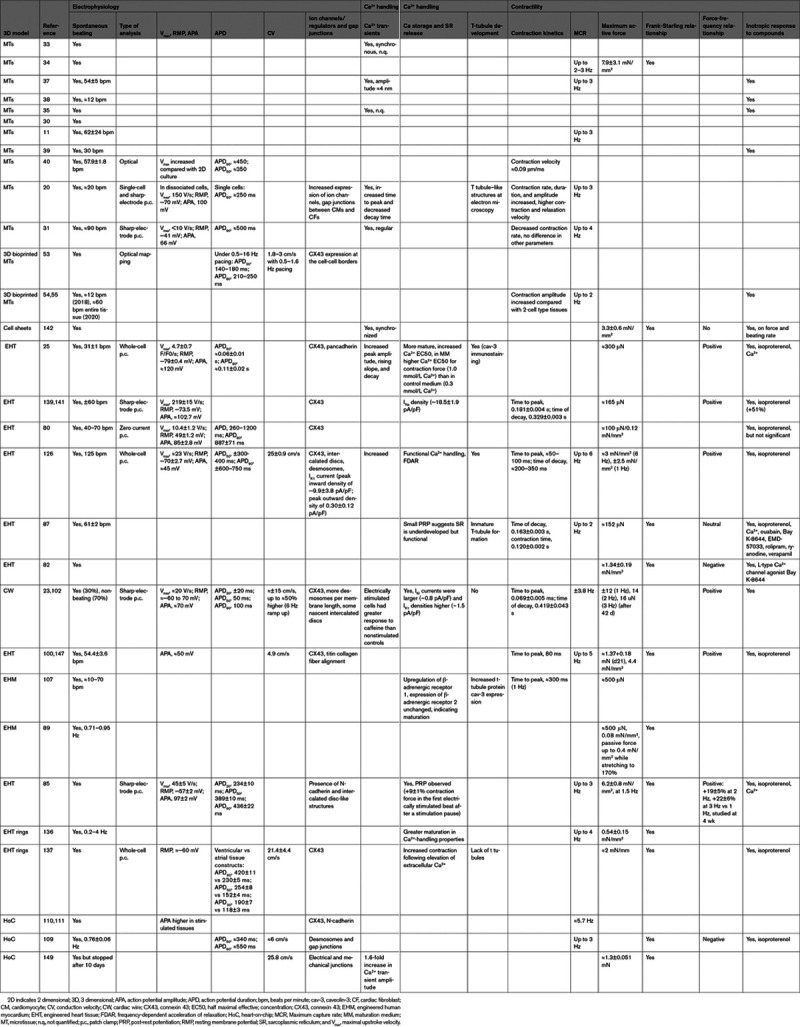
Electrophysiology, Ca^2+^ Handling, and Contractility in 3D Cardiac Tissues

Because they are easy to fabricate, amenable to high-throughput imaging and cell viability assessment, and their contractility is not dictated by physical properties of the substrate, cardiac microtissues are being used to investigate structural cardiotoxicity of drugs not only directly on cardiomyocytes, but also indirectly via the noncardiomyocytes also present.^44^ Cardiac microtissues have also been used as models to study MI: cell viability decreased in microtissues following hypoxia (10% O_2_), accompanied by sarcomere and mitochondria disorganization and altered secretion of cytokines, which recovered during the postischemia to postreperfusion transition.^45^ Three cell type microtissues exposed to hypoxia in another study showed an outside-to-inside oxygen gradient causing the center of the spheroid to become apoptotic, again mimicking MI. This was accompanied by differential gene expression, impaired oxidative metabolism with increased fibrosis that led to aberrant calcium (Ca^2+^) handling, similar to that in the native human heart after MI. Of note, the effect on Ca^2+^ handling was not observed in microtissues without fibroblasts.^46^ We recently used 3-cell type microtissues to model ACM—a rare genetic cardiac disease characterized by arrhythmia followed by formation of fibrofatty deposits. Inclusion of hiPSC CFs from ACM patients in cardiac microtissues was sufficient to induce arrhythmia during pacing, even when the hiPSC cardiomyocytes and ECs in the microtissues were from healthy controls. CX43 (connexin 43) gap junction expression was decreased in the microtissues, suggesting that defective cardiomyocyte-CF communication could contribute to arrhythmia in ACM patients.^20^

#### 3D Bioprinted Cardiac Tissues

3D bioprinting using bioink composed of cardiac cells (and hydrogels) can be used to produce larger cardiac tissues, rapidly and in large numbers. The challenge, however, is to minimize cell stress during printing and provide close-to-native cardiac ECM.^47^ Bioprinting methods have recently been reviewed^48–51^ but in essence started using rat or hiPSC cardiomyocytes with hydrogel bioinks made from collagen, alginate, gelatin, or fibrin printed in the form of cardiac patches.^50,51^ Scaffold-free bioprinting is also feasible and provides better biocompatibility for transplantation to the heart. Bioprinting has the advantage over microtissues that complex tissue architectures with specific cell organization can be created. Although cells for bioprinting can be added to the construct individually, most often they are first preassembled as microtissues so that they produce their own ECM giving the construct greater stability. This method is faster than single-cell and hydrogel printing, but ECM production can be insufficient or variable.^52^ For the first bioprinted microtissues with hiPSC cardiomyocytes, ECs, and fibroblasts together, vacuum suction was used to load individual preassembled microtissues containing 33 000 cells onto a needle array, such that they overlapped and fused, forming a large, scaffold-free tissue. The constructs engrafted and were vascularized after implantation in nude rat hearts.^53^ Tubular scaffold-free constructs have also been built in a similar way using multiple layers of 3-cell type microtissues^54^; these were sensitive to isoproterenol and propranolol and toxic effects of doxorubicin.^55^ Microtissues can also be printed layer by layer, which is especially useful for transplantation in vivo when larger constructs are required.^56^

Overall, 3D printing still requires the needle array, which acts as a support much like scaffold material; this increases prefabrication time and costs, and moreover, interactions of cells with the needle may interfere with cell-cell and cell-ECM interactions.

#### Cardiac Cell Sheets

3D cardiac cell sheets have been produced mainly for heart regeneration, since they preserve native ECM-cell interaction by omitting scaffolds. Cells can be detached as sheets from the culture dishes without use of enzymes by precoating the substrate with a temperature-responsive polymer, to which cells adhere at 37 °C but not at room temperature (20 °C). These sheets are then combined into multiple layers for direct use or transplantation into the heart.^57,58^ When thermoresponsive cell culture dishes coated with poly(N-isopropylacrylamide)^59^ were used to produce 3D cardiac tissues from rat cardiomyocytes, the sheets beat synchronously after detachment, established gap junction connections, and became electrically active and coupled with adjacent sheets.^57,60,61^ Elongated sarcomeres were observed in these tissues after transplantation into the subcutaneous space or hearts of nude rats.^62,63^

### Scaffold-Based Tissues

3D cardiac tissues have also been developed that deposit cells in scaffolds (Figure 1). These custom-designed biomimetic scaffolds recapitulate the geometric boundary conditions seen in vivo and thus guide cell self-organization into more physiologically relevant architectures. In the native heart, preload is the stretch during chamber filling, while afterload is the pressure the heart must work against to eject blood; these can be mimicked by anchoring or stretching the tissues, respectively. Indeed, tissues can contract unidirectionally against a load determined by scaffold properties (material stiffness or the presence of fixed attachment points like pillars). The scaffold properties can be used to calculate the force generated by the cardiac tissues by (1) measuring the displacement of points to which the scaffold is attached, (2) calculating the force required for the displacement knowing the material properties, and (3) dividing this force by the cross section of the tissue to obtain force per square micrometer. Scaffold mechanical and structural support maximize the viability of cardiomyocytes, which align in the direction of contraction. Various hydrogel mixtures have been used (collagen, Matrigel, and fibrin proteins); all can affect cell migration, organization, and functionality by engaging different cell surface integrins (ECM receptors) or having variable shear moduli. Engineered tissues have been formed in 2D or 3D formats as muscle thin films (MTFs), cardiac wires (CWs) or engineered heart tissues (EHTs) (Figure 1). We consider each of these individually below.

#### Muscular Thin Films

MTFs enable topological control of cardiomyocyte cultures as the cells are plated on elastomer thin films micropatterned with ECM proteins. The ECM patterns are typically arrays of lines with widths ≈15 to 115 µm and spaces between the lines ≈20 to 25 µm. The cardiomyocytes align and form anisotropic monolayers,^64–66^ which facilitate synchronized contraction, and because the MTF is fixed at one end, it curls up during systole and flattens during diastole^66^; this displacement can be measured using high-speed, high-resolution imaging, and knowing the material properties and geometry, the force generated can be calculated.^67^ MTFs are usually made of polydimethylsiloxane (PDMS) using microfabrication techniques. The load on the cardiomyocytes can be altered by changing the substrate thickness.^67,68^ Microfabrication allows actuators, electrodes, and force sensors^69^ to be embedded in the MTF, enabling, for example, electrical field stimulation of the tissue.^70^ Microcontact printing can be used to deposit ECM proteins in any geometric pattern on PDMS, with cardiomyocytes adopting the same shape as the patterned matrix. First, MTFs used rat cardiomyocytes,^71^ but later, hiPSC cardiomyocytes were used.^72^ Of note, neonatal rat cardiomyocytes remodeled shape and cytoskeleton within 48 hours, faster than adult cardiomyocytes,^73^ but remain functional for weeks.^72^ Two studies used MTFs to model mitochondrial cardiomyopathy^74^ and catecholaminergic polymorphic ventricular tachycardia^75^ and found that reactive oxygen species scavenging might benefit Barth syndrome patients and abnormal calmodulin-dependent protein kinase II–dependent reentry plays a role in catecholaminergic polymorphic ventricular tachycardia. Apart from standard multiwell plate formats, MTFs can also be included in 3D microphysiological system environments such as a fluidic channel or an endothelial barrier insert.^70,76,77^

#### Engineered Heart Tissues

EHTs were first developed using neonatal or embryonic cardiomyocytes from rat or chicken heart.^78,79^ EHTs based on hydrogels, containing cardiac cells cast in a mold with 2 elastomeric pillars, are now widely used. The cells self-organize as the hydrogel compacts and forms a tissue bundle around the two pillars, which exert continuous mechanical strain promoting cell alignment and thus auxotonic contraction of the tissue. From pillar deflection, optical readouts of contractile parameters (frequency and contraction and relaxation times) can be obtained and force can be calculated.^80–82^ Even early EHTs showed a high degree of longitudinal orientation, intercellular coupling, and force generation,^78,83^ and drugs like chromanol, quinidine, erythromycin, and doxorubicin exerted expected responses.^84^ Later, using hPSC cardiomyocytes, often in combination with skin fibroblasts,^85^ EHTs were also shown to exhibit expected physiological responses to cardioactive stimuli^86^ and drugs,^87^ outperforming 2D hPSC cardiomyocyte models and isolated rabbit cardiomyocytes in predicting response of inotropic drugs.^22^ Moreover, in this particular study, EHTs were the only model—apart from the isolated rabbit cardiomyocytes—that showed a positive force-frequency relationship (FFR). Ventricular hPSC cardiomyocytes have mostly been used to build EHTs, but this has recently been extended to atrial cardiomyocytes. Right atrial hPSC-EHTs had faster contraction kinetics, lower force generation, shorter action potential duration, and higher repolarization fraction than ventricular-like EHTs.^88^

Engineered human myocardium (EHM) is similar to EHT but is generated by integrating the cell-gel mixture with nylon mesh tabs instead of pillars.^89^ In addition to static stress and electrical pacing, uniaxial tension can also be exerted. Sarcomeres appeared more aligned under static stress compared with no stress or 1 Hz/4 days of cyclic stress.^89^ Cord-like structures, some containing lumens, were observed when ECs were added to the EHMs, and these increased markedly when stromal cells (mouse embryonic fibroblasts or human bone marrow stromal cells) were also added.^89^

More complex EHT designs can include a single implanted platinum wire to induce more physiological activation of a propagation wave in the tissue, rather than whole-field stimulation with 2 electrodes that activate all cells in the tissue simultaneously.^90^ EHTs as rings, where tissues compact around a central rod,^91^ provide EHTs without anchoring zones, thus isometric contraction (contraction without change in length); of note, although easier to reproduce, isometric contraction is less physiological than auxotonic contractile work on elastomeric pillars.^83^

Both types of engineered tissues have been used for modeling cardiomyopathies, since they are particularly suited to measuring changes in contraction force associated with these conditions. For example, dilated cardiomyopathy,^92–94^ hypertrophic cardiomyopathy,^85,95–98^ and familial cardiomyopathy^99^ have been investigated. The majority of the studies included mutant cardiomyocytes in EHTs, while one study induced hypertrophy by chronic β-adrenergic stimulation.^85^ Cell interactions that take place in myocardial interstitial fibrosis were investigated by including excess pericytes; the study showed fibrotic tissue responses marked by decreased contractility, increased tissue stiffness, secretion of BNP, and upregulated myofibroblast-associated genes.^100^

In general, casting cardiac cells in hydrogels results in highly reproducible cardiac tissues with unidirectional force of contraction. EHTs and EHMs thus represent a valuable tradeoff between the fully controlled MTFs and self-assembled scaffold-free cardiac tissues.

#### Cardiac Wires

CWs were developed to provide both structural cues and electrical field stimulation with view to enhancing hiPSC-cardiomyocyte maturation. CWs also use cell-hydrogel mixes but can be cast in PDMS channels with an anchored surgical suture in line with the channel.^101^ An updated version has 2 parallel poly-octamethylene maleate (anhydride) citrate wires fixed at both ends of the microwell.^102^ In both versions, the tissue experiences high longitudinal tension, which facilitates uniaxial contraction and alignment. Displacement of the wires enables quantification of passive tension and active force, but Ca^2+^ transients and electrical properties can also be measured.^23,101^ Cardiomyocytes in CWs also show canonical responses to compounds that act via β-adrenergic/cAMP pathway (isoproterenol and milrinone), L-type Ca^2+^ channel (FPL64176 and nifedipine), or indirectly affect intracellular Ca^2+^ concentrations (digoxin). Positive inotropic drugs induced expected responses in these tissues.^103^ Additionally, CWs have been used to model left ventricular hypertrophy.^102^ While most engineered myocardium generated from hPSCs is ventricular like, heteropolar cardiac tissues containing distinct atrial and ventricular ends have also been developed (atrioventricular CW).^102^ Perfusion through the wire mimics vascularization of the heart.^104^ Fibrosis was also modeled by increasing the CF content and using a fibrin-based hydrogel to encapsulate the cells. This enabled quantification of collagen deposition, which is increased in fibrotic myocardium.^105^ These heterogeneous CWs allowed the effects of antifibrotic compounds to be examined on equivalents of the scar lesion, border zone, and adjacent healthy myocardium simultaneously, with convenient functional readouts.^105^ A variant I-wire system enabled the culture of 3D cardiac tissues between titanium wires to form an elongated cardiac muscle.^106^ Here, an integrated flexible probe provided strain loading via lateral displacement. Passive tension and active contractility can be quantified by optical measurement (fluorescent probe or tissue displacement), and the tissue can be electrically stimulated or subjected to sustained mechanical load.^107^

#### Microfluidic Platforms

Organs-on-Chips, where tissues are integrated in engineered microfluidic chips, now include heart-on-chip modalities. Microfluidic channels lined with ECs can mimic blood vessels and the crucial dialogue between cardiomyocytes and ECs undergoing blood flow or interaction with inflammatory cells and cytokines.

Pulsatile tubular cardiac tissues were generated with cell sheets containing hiPSC cardiomyocytes and human dermal fibroblasts and then wrapping these sheets around a flexible octagonal column. Small slits on the sides of the column in these bioreactors allowed pulsation and perfusion (0.5 mL/min). Spontaneous beating or electrical stimulation of the tissues resulted in electrical and inner pressure changes.^108^ Mimics of pumping heart chambers were also developed that were uniquely able to measure pressure and volume metrics, and pressure load could be controlled with an adjustable hydrostatic fluid column.^109^ In another approach, the gel-cell mix was seeded between multiple microposts to form a 3D tissue flanked by 2 medium channels; an integrated pneumatic actuation system induced homogeneous uniaxial cyclic strain to the tissue.^110^ Incorporation of electrodes allowed mechanical and biochemical costimulation of this heart-on-chip.^111^ A microfluidic device with 4 compartments was also developed. It consisted of a microchannel, chamber, diaphragm, and a push bar that controlled the volume of the microchannel. Displacement of the beating tissue, measured using fluorescent particles in the microchannel, indicated positive FFR in this device.^112^

## Scaffolds and Matrix: Do These Matter?

In contrast to conventional (2D) cultures, which are typically on rigid polystyrene (PS), scaffold-based tissues are usually cultured on substrates that have mechanical properties more closely resembling those of the cellular niche, such as silicone rubbers or gels. The most widely used material for this purpose is PDMS, which is easy to manipulate, gas-permeable allowing oxygen diffusion, optically transparent, and biocompatible. However, while PS is far from inert, PDMS is notorious for drug absorption, which was thought to be related to compound hydrophobicity but is more likely related to its topological polar surface area.^113^ As an example of how PS and PDMS differ, the concentration of cardioactive drug bepridil after 3 hours in the culture medium was reduced by ≈50% by tissue-culture PS but over ≈80% by PDMS.^113^ A particularly useful feature of PDMS in cardiac tissue engineering, however, is its availability in different stiffnesses and elasticities allowing changes in cardiomyocyte (after)load and thus the force generated.^21^ In contrast to PS with a fixed Young modulus of ≈3.7 GPa,^114^ PDMS stiffness can be tuned within a physiological range,^115^ allowing healthy (load, <50 kPa) or diseased (eg, fibrotic; load, ±100 kPa) cardiac environments to be modeled.^116^ More instantaneous changes in afterload during culture can be achieved by inserting metal braces in the pillars of EHTs. This has been shown to induce cardiomyocyte enlargement of 28.4% in neonatal rat tissues—a hallmark of pathological hypertrophy.^117^ Another approach to tuning pillar stiffness uses magnetic beads.^118^ Translational applications, especially those concerning disease modeling, can benefit from use of these more complex, physiologically relevant substrate materials, but difficulty in predicting the extent to which they can reduce compound bioavailability still limits their use in pharmaceutical screening pipelines.

Besides scaffold material, ECM also plays an important role in determining the (patho)physiological nature of the model, since this fibrous protein network provides a biological niche for tissue assembly and its physical features govern cell behavior. ECM is known to closely interact with cells by presenting growth factors to their receptors and sensing and transducing mechanical signals, which influences cell growth, morphology, function, migration, survival, gene expression, and differentiation.^119^ In scaffold-free cardiac tissues, ECM is self-secreted, but in scaffold-based tissues, it is mostly added exogenously. Differences in chemical and mechanical properties contribute to cardiomyocyte alignment, intracellular organization, and force in seeded tissues.^23,120,121^ While it has not yet been possible to rely on ECM self-secretion to form cardiac EHTs, this might be a way forward ensuring only physiologically relevant ECMs are actually present.

To control and manipulate stiffness in vitro, ECMs can be electrospun to align the fibers or the hydrogel concentration can be changed.^122^ There are now even methods that allow ECM or hydrogel stiffness to be modulated during cell culture by temperature or light.^123^ There are many ECM types in humans, of which collagen, fibronectin, and elastin are most common in the healthy human heart.^124^ These are present in precisely controlled ratios that may be altered in disease states. For example, collagen I:III ratio is ≈0.6 in healthy hearts but ≈1.2 in dilated cardiomyopathic hearts.^125^ Careful selection and control of the composition of ECM might, therefore, be important for disease modeling in vitro. Nevertheless, many cardiac tissue engineering approaches do not take this into account and even use ECMs not present in the healthy heart but, like fibrin,^126^ for example, only formed during scaring after MI. The composition and mechanical but also thermal properties of the ECM are also extremely important when used for 3D bioprinting as these characteristics determine the resolution achievable in the bioprinted models. Moreover, in some cases, material properties can affect cell viability due to the extrusion process through the nozzle.^127^ When engineered cardiac tissues are used for heart regeneration, scaffold and ECM need to be compliant with good manufacturing practice. Clinical-grade ECMs are available but are typically expensive although might be replaced by engineered ECM.^128^ For cotransplanted scaffold carriers for the cardiac tissues, biodegradable materials also offer solutions.^129^

## Maturation in 3D Cardiac Models

Immaturity is an important shortcoming of hPSC cardiomyocytes for modeling cardiac disease or drug responses in adults since ordinarily they do not develop further than the equivalent of 16 weeks for human gestation.^130^ One motivation for developing 3D cardiac tissues was to increase the maturation state of hPSC cardiomyocytes so that their functional and structural properties are more like native adult tissue. This means increased contraction force, positive FFR, post-rest potentiation (PRP) and Frank-Starling law, greater sarcomere organization and length, electrical coupling through gap junctions, higher mitochondria number and alignment with sarcomeres, mitochondrial cristae development, the presence of transverse tubules, and metabolic switch from glucose to fatty acids. Although in many studies, relative rather than absolute values for these parameters are reported, the overall degree of maturation in any 3D cardiac model is improved compared with standard 2D culture even though none have yet achieved that of adult cardiomyocytes. In some cases, maturation has been promoted not only by the 3D environment but also by reproducing (via external stimulation) the mechanical load and the electrical pacing regime that cardiomyocytes experience in the heart. Mechanical load can result in auxotonic contraction (more physiological, most like that in EHTs^131^) or stretch in the tissue. Afterload also plays an important role in functional maturation of cardiomyocytes in EHTs.^117,132^ While moderate afterload promotes cardiomyocyte maturation, further increase may be detrimental and cause pathological changes.^133^ Here, we discuss in more detail maturation in structure, function, and metabolism in the different models.

### Structure

Many scaffold-based 3D models and some scaffold-free 3-cell type cardiac microtissues achieve sarcomere lengths and organization similar to adult cardiomyocytes.^134,135^ In general though, scaffold-free cardiac tissues have been analyzed in less depth than scaffold-based tissues (Table 1).

Microtissues showed increased myofibrillar organization over time when hiPSC-cardiomyocyte purity was <50% at the beginning of 3D culture. This was an early indication that undefined noncardiomyocyte cells present may affect initial aggregation and maturation.^33^ Microtissues containing hESC cardiomyocytes, HUVECs (human umbilical vein endothelial cells), and mouse embryonic fibroblasts indeed showed enhanced collagen production and mechanical stiffness.^34^ Subsequent studies on 1/2/3-cell type microtissues found structural maturation in scaffold-free microtissues, with enhanced ECM (collagen, fibronectin, and laminin) deposition^30,35,40^ and increased expression of the mature isoforms of sarcomeric proteins.^20,31,35,37,38^ We also showed enhanced sarcomere organization and length using cardiomyocytes, ECs, and CFs from hiPSCs concurrently in 3-cell type microtissues.^20^

The first (large) rat EHTs^78,79^ and later (smaller) hESC cardiomyocyte/fibrin-based EHTs with fewer cells similarly showed enhanced cardiomyocyte alignment and well-developed sarcomere organization. CX43 junctional protein expression was evident although not confined to intercalated discs as in adult heart.^80^ Structural maturation was also observed in EHMs, reflected in an increase in the MYL7:MYL2 ratio, cardiomyocyte alignment, and (physiological) hypertrophy.^85^ Using hiPSC cardiomyocytes expressing the genetically encoded Ca^2+^ indicator GCaMP3, looped activation propagation was found in EHT rings, together with improved structural maturation and enhanced adult cardiac gene expression in a frequency-dependent manner.^136^ After transfer to a silicon stretcher, both ventricular- and atrial-like EHT rings underwent similar structural and functional maturation.^137^ In EHMs subjected to passive stress, an optimal stretch distance resulted in aligned cardiomyocytes, mechanical contractions throughout the whole tissue, coordinated Ca^2+^ waves, and upregulation of genes associated with maturation without the need for cyclic stretch.^107^

Long-term electrical stimulation for cardiomyocyte maturation was first reported in nonhuman EHTs.^79,138^ Biphasic pacing (1 week at 2 Hz, the next at 1.5 Hz) for 4 days after EHT formation improved sarcomere ultrastructure, with regular M bands, increased CX43 abundance, and improved external Ca^2+^ response. When pacing was increased to supraphysiological levels for 3 weeks, much greater maturation was reported with sarcomere lengths of ≈2.2 µm and strikingly mature features in histology.^126^ However, this report has recently been extensively corrected and has yet to be reproduced by others. Interestingly, inflammatory gene expression was detected in stimulated EHTs, likely due to oxidative stress caused by the electrical pacing.^139,140^ Also in CWs, increasing pacing improved ultrastructural organization (frequent myofibril convergence with sarcomeres, aligned Z discs, numerous mitochondria, desmosomes, many H zones per sarcomere and I bands per Z disc) and increased cardiomyocyte size.^23,101^

### Function

Notably, all models still show spontaneous beating, with depolarized resting membrane potentials compared with adult cardiomyocyte (≈−90 mV) and other immature functional features (Table 2). However, the beat rates in microtissues and EHTs vary between studies, ranging from 20 to 80 bpm (Table 2).

Electrophysical properties of cardiomyocytes in 3D cardiac tissues are technically difficult to measure but can be analyzed using impaling electrodes or first dissociating the tissue into single cells for conventional patch-clamp analysis. Voltage-sensitive dyes have also been used although they are less sensitive and do not provide absolute measures of all parameters. Different techniques and cellular components in microtissues have led to a wide range of electrophysiology values being reported. For example, voltage-sensitive dyes in cardiomyocyte-only microtissues showed increased action potential duration and upstroke velocity^40^ (Table 2), while sharp-electrode patch clamp in microtissues with CFs and hiPSC cardiomyocytes showed rather slow upstroke velocities (66 V/s) and depolarized (more positive) resting membrane potential (−41 mV)^31^; 3-cell type microtissues by contrast showed longer action potential duration, hyperpolarized resting membrane potential, and faster upstroke velocities, independent of whether measured by patch clamp in whole-tissue microtissues or as single cells after dissociation.^20^

Electrophysical properties in EHTs were typical of more mature cardiomyocytes, with similar I_Na_ density and upstroke velocities as in human ventricular tissue.^25,80,126,141^ Recently, I_to_ notches were reported in the AP profile of CWs measured with sharp-electrode patch clamp.^102^ The positive PRP in hPSC-EHTs, albeit small, indicated the capacity of the sarcoplasmic reticulum to store and release Ca^2+^.^87^

Contraction kinetics of microtissues and EHTs also differed quantitatively between studies (Table 2). Multicell type composition promotes functional maturation, as cardiomyocytes in 3-cell type microtissues showed enhanced contractile function (higher beating rate and Ca^2+^ transient amplitude) and expected responses to ionotropic drugs, through upregulation of sarcomeric and calcium-binding proteins, compared with cardiomyocyte-only 3D culture.^37^

To assess contractility, conduction velocity (CV) and contraction force are informative parameters, but they can be measured only in some (mostly scaffold based) models. In 3D printed microtissues, CV and contraction force have been assessed using voltage-sensitive dyes. CV was relatively slow (≈2–3 versus 30–100 cm/s in adult human heart),^53^ but contraction force increased with time in culture, although could not be measured absolutely since the needle tip was not microscopically uniform.^54^ One study measured contraction force in cardiac cell sheets by placing sheets onto fibrin gels in a customized device.^142^ This showed contractile force around 1 mN and 3.3 mN/mm^2^ of cross-sectional area, higher than in some other models but still far from that of the adult human heart (40–50 mN/mm^2^).^143^ The study showed positive Frank-Starling relationship but negative FFR, again indicating some but not complete maturation.^142^ Cell sheets do allow cell alignment by micropatterning of the cell culture surface^144^; this may also improve cardiomyocyte contractile function.

In EHMs, the force of contraction increased to 6.2±0.8 mN/mm^2^ at 1.5 Hz^85^ after 8 weeks of culture, which is higher than the force of contraction in papillary muscle from human infants (≈1 mN/mm^2^ at 1 Hz)^145^ but still lower than in healthy adult myocardium. Interestingly, the force-length relationship was similar to the Frank-Starling curves in intact heart.^85^ Nevertheless, it was claimed that these EHMs only resembled the 13-week human fetal heart.^85,89,107^

Electrical stimulation (5 V/cm, 2 Hz, 5 ms) in combination with static stretch causes cell alignment, physiological hypertrophy, increased force (1.34±0.19 mN/mm^2^), and Frank-Starling–like force-length relationships. In addition, functional maturation associated with enhanced expression of sarcoplasmic reticulum–related proteins SERCA2 (sarco-endoplasmic reticulum calcium ATPase 2) and RYR2 (ryanodine receptor 2) was observed.^82^ Stepwise increases in cyclic stress of EHM cultured between 2 titanium rods resulted in improved sarcomere alignment, cardiomyocyte coupling, and active tension (4.4 mN/mm^2^), claimed to be comparable with native myocardium.^146,147^ Higher contraction force was reported after biphasic pacing in EHTs; however, it was still lower (≈0.23 mN) than in adult cardiomyocytes, mitochondria were immature, and the sensitivity to Ca^2+^ remained too high.^139^ Pacing at supraphysiological levels produced positive FFR,^126^ characteristic of human adult ventricular myocardium, but also seen in earlier EHT models.^85^ Since positive FFR might be masked by increased funny current (I_f_) that triggers spontaneous contraction in hPSC cardiomyocytes, the I_f_ blocker ivabradine has been used to induce quiescence before measuring FFR, which is then investigated by pacing the tissue at lower frequencies (0.75–2.5 Hz), which revealed a positive FFR.^86^

Several studies indicated that hPSC cardiomyocytes in CWs respond much the same way to pacing as EHTs: gradual increase toward 6 Hz produced more mature CV, electrophysiology, and Ca^2+^ handling.^23,101,126^ The authors hypothesized that 6 Hz stimulation (twice that of the human fetal heart) could be a compensatory mechanism for the lack of noncardiomyocytes, indirectly resulting in cardiomyocyte maturation.^101^ Addition of increasing numbers of CFs in CWs increased passive tension and decreased active force. Moreover, adding fibrin to the collagen hydrogel improved intracellular organization of cardiomyocytes. These tissues showed adult-like functionality and maturation: positive FFRs, substantial PRPs, and active force measurements up to 16 μN.^23^ In blinded validation, drug-induced changes on CWs were examined. Inotropes with different mechanisms of action (including β-adrenergic agonists, PDE3 inhibitors, Ca^2+^ sensitizers, myosin and troponin activators, and an apelin receptor agonist) could be distinguished, adding this to the models that may be predictive for new therapeutics.^148^ Interestingly, robust positive FFR, PRP, and fast CVs were observed, although these were still below that of adult myocardium (30–100 cm/s).^102^

Microfluidics was also shown to improve functional maturation in heart-on-chip models. Dynamic conditions (platform rocker, 30° tilt, 0.4 Hz) yielded 2.5-fold higher active force compared with static conditions, physiological Frank-Starling mechanism, increased cardiomyocyte density and hypertrophy (Table 2).^149^ Biomimetic systems using a pressure gradient to move fluid through the cardiac system as in the native heart showed that physiological mechanical loads and biaxial stretch did not substantially improve cardiomyocyte maturation.^150^ In contrast, cyclic stretching (5%), electrical stimulation (1 Hz), and vascular perfusion of 0.2 mL/min did improve maturation.

### Metabolism

Metabolic maturation is rarely reported in 3D cardiac models. Two studies using microtissues and different methods to assess metabolism showed energetically more efficient glucose metabolism, with reduced glycolysis and enhanced mitochondrial oxidative phosphorylation.^20,40^ Most EHTs have been cultured in glucose-based media, mimicking the metabolic state of fetal hearts.^151^ However, culture medium with low carbohydrates, low insulin, no serum, and palmitate changed EHT metabolism toward fatty acids and also induced some degree of maturation.^25^ Passive stretch has also been shown to cause a similar metabolic switch in hiPSC cardiomyocytes from EHTs.^152^ Key proliferation pathways (β-catenin and Yes-associated protein 1) were repressed, force increased, Ca^2+^ handling was more mature, mitochondrial mass was increased, and overall organization improved^25^ (Tables 1 and 2).

## How 3D Cardiac Tissues Could Be Used in Regenerative Medicine

**Figure 2.**
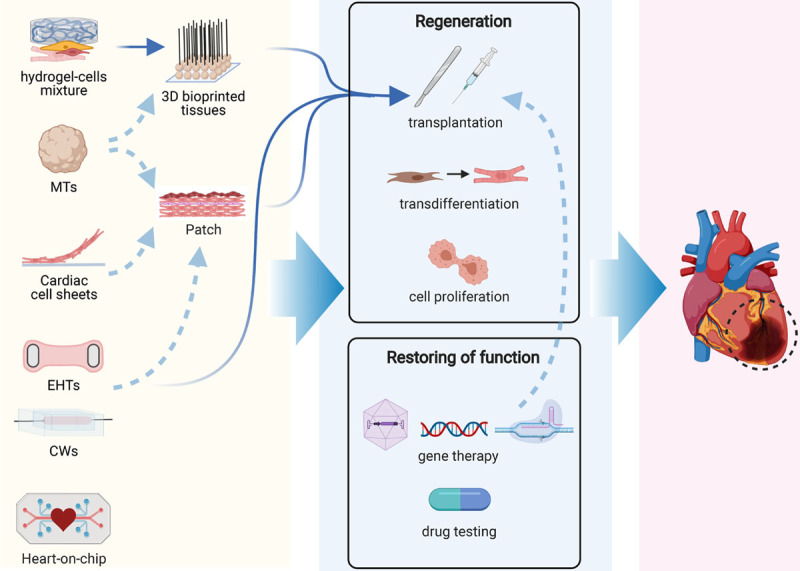
**Three-dimensional (3D) cardiac models in regenerative medicine.Left**, Overview of models applicable to cardiac regenerative medicine: microtissues (MTs), cardiac cell sheets, engineered heart tissues (EHTs), and cardiac wires (CWs) can used for transplantation either alone or as cardiac (3D bioprinted) patches. **Middle**, Applications of models in cardiac regenerative medicine, including tissue regeneration and restoration of tissue function. Gene therapy can be also used in addition to or instead of transplantation. **Right**, The ultimate goal is to restore function of damaged hearts in vivo.

The human heart is limited in its capacity to regenerate since it appears to lack cardiomyocyte stem cells and adult cardiomyocytes divide only slowly.^153^ Repair after damage is then largely through scar tissue formation. Restoration of healthy myocardium to maintain proper contractile function and conductivity is essential to prevent subsequent heart failure or arrhythmogenic behavior, respectively, and various approaches are currently being investigated for this purpose (Figure 2). They include directed transdifferentiation of CFs to cardiomyocytes in situ^154^ and replacement of cardiomyocytes by transplantation of hPSC cardiomyocytes.^3^ Unless donor derived, hPSC cardiomyocytes would require lifelong immunosuppression to prevent rejection. In addition, both direct reprogramming and ectopic cardiomyocyte transplantation would need careful control of potential arrhythmogenic risk and ways to ensure that sufficient but not excessive cardiomyocyte numbers are present in the heart. First transplantation studies of hPSC cardiomyocyte suspensions into the heart were performed in rodents, later in nonhuman primates.^155–160^ Cell delivery was intracoronary, intravenous, or intramyocardial. This was reported to attenuate cardiac remodeling,^161^ improve vascularization,^162^ and (transiently) improve cardiac function.^156,163,164^ Absence of electrical coupling with host tissue^155^ was always a limitation, sometimes causing malignant ventricular arrhythmia.^162,165^ Poor retention and survival of hPSC cardiomyocytes was often observed,^166,167^ although scaffold embedding with prosurvival factors prevented apoptosis after transplantation, resulting in low engraftment efficiency.^161^

An alternative cardiac regeneration and repair that could overcome these limitations is the use of multicell type 3D cardiac (engineered) tissues containing hPSC cardiomyocytes for transplantation (Figure 2). As hPSC cardiomyocyte suspensions, teratomas were not observed after transplantation of (engineered) tissues,^168^ and these could be constructed without animal components, paving the way for good manufacturing practice compliance in humans.^82,85,168^ Specialized catheters were required for delivery^169,170^ and, for larger tissue constructs, open chest surgery.^171,172^ In another approach, cell sheets of nonhuman primate ESC-derived SSEA-1^+^ cells (described as cardiac progenitors) and adipose tissue–derived stromal cells were transplanted into the hearts of primates where they engrafted and differentiated in situ into cardiomyocytes.^173^

One of the first studies to restore myocardial function in vivo in rat hearts used collagen-based EHTs from neonatal rat cardiomyocytes.^174^ Although hPSC-derived cardiomyocytes in EHTs and EHMs survived and engrafted (with up to 25% of the transplanted cells retained until week 12),^168^ ultimately there was no functional benefit compared with cell free controls.^168,175,176^ Lack of electromechanical coupling or vascularization is often the cause. Electromechanical coupling was seen in microtissue-based size-controlled (through number of input microtissues) patches,^33^ which, more generally, can be constructed from different cardiac cell types and used for direct injection or as building blocks to generate tissue engineered constructs.^35^ When bioprinted 3D cardiac tissues (2×2 cm×400 μm) composed of human fetal heart SCA1^+^-derived cells in hyaluronic acid/gelatin-based matrix were transplanted epicardially onto ischemic mouse hearts, engraftment and long-term survival were observed.^177^ These printed cardiac patches resulted in smaller scars, improved cardiac function, and better cardiomyocyte survival compared with the control group.^178^ Cardiac patches can also be printed using microtissues containing hiPSC cardiomyocytes, ECs, and fibroblasts, without biomaterials, and following transplantation to rat heart, they become vascularized from the host.^53^ Enhanced cardiac function was observed in rat hearts after MI first using rat cardiomyocyte–derived cell sheets^179^ but later using either hiPSC cardiomyocytes alone^180–182^ or hiPSC cardiomyocytes with hiPSC vascular cells (ECs and mural cells).^183,184^ hiPSC cardiomyocytes were also used in combination with hiPSC ECs in transplantable EHTs, and these improved left ventricular function after implantation in MI guinea pig hearts.^175^ The importance of multiple cell types was also seen in microtissues containing cardiomyocytes, CFs, and ECs on a fibrin-based patch, which even showed vascularization after positioning over the infarcted region of the mouse heart, electromechanical coupling, and over 25% engraftment; this resulted in improved cardiac function in vivo in mouse hearts.^185^ Multilayered cardiac cell sheets consisting of different cell types to form patches also facilitated microvessel sprouting into the host cardiomyocyte layer and coupling with the host vasculature improving cell survival and maturation.^184,186^ More recently, multicell type bioprinted constructs containing hiPSC cardiomyocytes and HUVECs encapsulated in hydrogel strands with alginate and PEG-fibrinogen were shown to improve integration in vivo.^187^

Optimization of the grafts in vitro using similar approaches as described for tissue maturation is promising to improve 3D constructs for translation. Cyclic stretch, electrical stimulation, and vascular network formation^188^ in hPSC-derived EHTs before engraftment increased contractile and intercellular organization and may facilitate perfusion by the host coronary circulation,^189^ evidenced by host erythrocytes in the constructs.^89^ Importantly, hiPSC-derived EHTs were transplanted into guinea pigs after MI, and electrical activity was telemetrically monitored in the heart for 28 days. This showed there was no clinically relevant sustained ventricular tachycardia or ventricular fibrillation, suggesting electrical safety.^190^ Of note, this approach allowed the contractile performance of the EHT to be determined before implantation, which may benefit further optimization.^106^

Alternatively, the substrate of the patch can be engineered to facilitate coupling or vascular sprouting. For example, hESC cardiomyocytes were plated on to a prepatterned vascular bed of aligned microchannels,^36^ which induced neoangiogenesis and vascular remodeling.^36^ These perfusable grafts supported cardiomyocyte survival and integration with the host coronary vessels after transplantation in vivo into infarcted rat hearts much better than nonperfused constructs.^176^ Cardiac tissues cultured on these AngioChips and implanted to the femoral vessels of rat hind limbs in which ischemia had been induced underwent immediate blood reperfusion,^191^ suggesting that perfusable engineered microvasculature would promote formation of large vascularized 3D cardiac constructs suitable for regenerative therapy. Similarly, printed vascularized cardiac patches of 2- to 7-mm thickness, containing hiPSC cardiomyocytes and hiPSC ECs in hydrogel with blood vessel architecture optimized for oxygen transfer using mathematical modeling, showed elongated cardiomyocytes in vitro with striking actinin striation, high cell viability, and strong contractile activity.^192^ Printing small-diameter blood vessels within thick structures is challenging, but multiphoton 3D printing has been used to create native-like scaffolds from ECM, which contained hiPSC cardiomyocytes, ECs, and smooth muscle cells (SMCs) (ratio, 2:1:1). After transplantation to mice post-MI, contraction speed was increased and calcium handling improved.^193^

In summary, preformed 3D engineered multicell type (perfusable) cardiac tissues have the potential to form larger grafts than conventional approaches and may provide excellent opportunities for heart repair once severe damage and tissue loss has occurred. Approaches that exploit the potential of in vitro engineering and conditioning aimed at graft optimization for vascular and functional coupling in vivo are among the most likely candidates for safe and effective cardiac repair. The prospect of being able to remuscularize chronic myocardial scars and thus restore cardiac function in patients is far beyond cell transplantation ambitions and could even be performed in late-stage disease provided the surgery is feasible. Further studies that focus on functional coupling and vascularization in nonhuman primates and larger animals in this context are of high relevance.

### Gene Therapy

3D cardiac tissues based on human cells could also be used for testing the potential effectiveness of gene therapy (Figure 2): does a construct work in repairing the defective cell type, and how many cells need to be repaired to restore tissue function? This is most important for genetic conditions with now equivalent in animal models. The use of patient-specific hiPSCs in this context can contribute to the realization of personalized medicine. In addition, dedifferentiating adult cardiomyocytes in vivo to enhance reentry into the cell cycle followed by secondary redifferentiation may also be feasible. A proliferative barrier was reported to be imposed by fatty acid metabolism in EHTs and was rescued by simultaneous activation of β-catenin and YAP1 (yes-associated protein 1) using small molecules or genetic activation of these pathways.^25^

CRISPR-Cas9 gene repair has been used directly in 3D organoids although mostly on single cells before inclusion in the 3D tissue or after dissociation because delivery into 3D structures is still technically challenging.^194^ However, there are several recent examples of infection using viral vectors directly in 3D, for example, in retinal spheroids using adeno-associated viral vectors.^194,195^ This may eventually be used in heart models.

## How to Choose a Cardiac 3D Model

**Figure 3.**
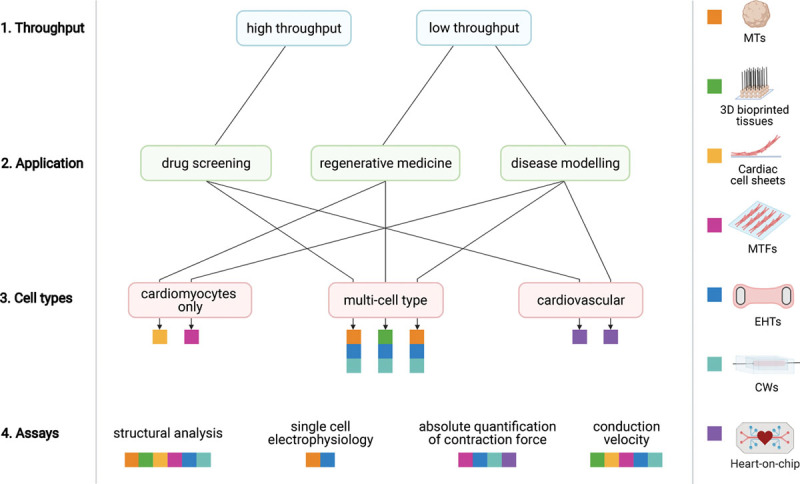
**How to choose a 3-dimensional (3D) cardiac model.** Key decision steps (**left**) are numbered in their order. Possible choice paths are indicated by arrows, leading to the different models, each represented by a different color, as indicated (**right**). CW indicates cardiac wire; EHT, engineered heart tissue; MT, microtissue; and MTF, muscle thin film.

Those new to the field may find it challenging to choose between the multiplicity of models now available for the human heart, all claiming to be true tissue mimics and to induce tissue maturation to a greater or lesser extent. As we mentioned earlier, a useful guiding principle is to keep a model as simple as possible to address the questions at hand. This means that it is not always necessary to actually rebuild native myocardium. Rather, a balance needs to be found between desired throughput, biological relevance, ease of use, time, cost, and meaningful readouts (Figure 3). This may include consideration of the numbers of cells required, the presence of scaffolds, or the requirement for specialized apparatus, all influencing the throughput. Scaffold-free microtissues self-assemble and meet requirements of simplicity and low cell numbers (2000–5000), reducing costs of each tissue so that their production can be inexpensively scaled. EHTs typically required large cell numbers making them costly, and they could only be produced in small numbers, limiting throughput, for example, in drug screens, or allowing only escalating dose to be measured on a single EHT rather than independent dosing. More recently though, EHTs have undergone miniaturization to 1µL- or 96-well formats^25,81^ using around 15 000 cells. EHTs have the advantage that the scaffold material (hydrogels, synthetic polymers with different viscoelasticities) can easily be modified, and electrical pacing and addition of mechanical load are feasible. The same is true for CWs, which also use few cells but may be more challenging to construct, and in some cases for cardiac patches (fused microtissues or cell sheets).^34,142^

Decisions on which models are most suitable are also dictated by the biological question or application (Figure 3; Table 3). For example, scaffold-free microtissues have significant benefits for (new) drug testing since many tissues can be produced in parallel at low cost and drugs can bind to the scaffolds. Several studies have assessed responses to positive and negative ionotropic drugs^11,20,35,38,39^ and cardiotoxins.^30,44,46,196,197^ These studies showed that many models can reproduce known drug effects on the heart. Even though throughput is presently lower and costs higher, EHTs have been successfully used for drug testing and are particularly appropriate for long-term measurement of drug responses, since they are more stable than scaffold-free tissues.^198^ Both microtissues and EHTs have also been used for disease modeling, as already mentioned, and changing the ratio of different cell types can mimic pathological heart conditions.^105,199,200^ Multicell type 3D constructs or microtissues that include both hiPSC cardiomyocytes and noncardiomyocytes^20^ are useful for modeling genetic cardiac diseases and investigating whether noncardiomyocyte cells contribute to or cause disease in addition to or instead of cardiomyocytes. This is most appropriate for precision or personalized medicine when all cells are derived from the same genetic background (pairs of genetically corrected and patient cells). Heart-on-chip models incorporate vascular-like fluidic flow through cardiac tissue and are of growing interest to study cardiovascular disease and drugs, where either passage of inflammatory cells or cytokines through the vasculature may affect responses of the heart tissue. However, their added value above simpler models (adding inflammatory cytokines alone) remains to be proven.

**Table 3.**
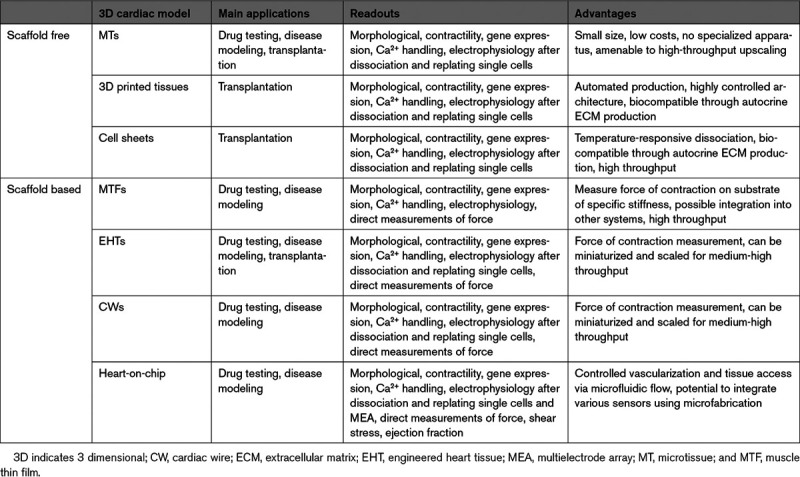
Applications, Readouts, and Advantages of 3D Cardiac Models

Finally, possible assays and readouts need also to be considered in the choice of the model (Figure 3). Absolute values are preferred as they allow comparison between independent studies, relevant to determining the severity of a genetic phenotype or drug responses. Among quantitative parameters that can be obtained for virtually all models are structural organization and contraction based on analysis of video recordings (Tables 1 and 3). Sarcomere organization in cardiomyocytes is fairly straightforward to measure in 2D formats but is more difficult in spheroid tissues like microtissues, where the cardiomyocytes are not precisely oriented. ECM produced in scaffold-free tissues or hydrogels in engineered tissues can make dissociation to single cells difficult and disrupt sarcomeres but when successful allows analysis by single-cell RNA sequencing or patch-clamp electrophysiology. We and others have demonstrated the feasibility of dissociation for microtissues and EHTs.^20,25,126^ Scaffold-based tissues allow direct measurement of force of contraction and CV, relevant for modeling conduction defects or cardiomyopathies, while this is not possible in microtissues (Table 2).

## Outlook: What Features Are Missing in Current Cardiac 3D Models?

Cardiac 3D models are coming of age in their ability to model healthy human myocardium for testing cardioactive drugs and are poised to contribute more widely to understanding cardiac diseases and even the interaction of the heart with other organs via linked organ-on-chip formats. Clearly, complete maturation and generation of all of the different cardiomyocyte and cardiac stromal cell subtypes found in the adult human heart^201^ is still pending, and it is essential to recapitulate the macrostructure of the heart more closely, as often required for modeling diseases.

In addition, knowledge about the interaction between cardiomyocytes and the vascular and lymph systems that may contain immune cells is also progressing. This would not only allow recapitulation of MI resulting, for example, from plaque formation, arterial blockage, or rupture (as in the study by Westein et al^202^), but also the 3 phases of cardiac repair that follow the arterial blockage.^191^ This may require development of methods to derive tissue-specific macrophages of the heart^203^ and for live imaging of these events through specific (genetic) reporters or sensors. Post-MI remodeling also includes sympathetic hyperinnervation, which can cause arrhythmias; models of this process could be useful in finding alternative treatments to surgical section. Interaction with the liver mediates responses to cardioactive drugs, where some molecules need to be metabolized before becoming functional or, alternatively, are inactivated by the liver before being effective. Linked heart- and liver-on-chip models may provide a solution when fully developed.^204^ Finally, the lymph system is important for fluid drainage after infarction, and better understanding of how this works through models that include lymph vasculature may identify new ways of treating cardiac edema. Congenital heart defects are usually caused by abnormal patterning of the heart after cell differentiation has been initiated, and models that could capture linear heart tube formation, looping, and chamber formation and regionalization^205^ would be a useful addition to understand how these defects occur. The rapidly developing area of gastruloids may be one to fill this gap: gastruloids derived from mESCs can form relatively advanced stages of gastrulation with anterior-posterior axis formation and left-right asymmetry, which could provide a model for studying ventricular inversion or transposition of the great arteries in situs inversus, should it be possible to achieve similarly advanced stages with hESC or hiPSC.^206–208^

The ability to translate any data derived from cardiac 3D models to the patients is important to demonstrate usability and clinical relevance. Typical measures for assessing cardiac health clinically are ejection fraction output using echocardiogram, electrical activity using ECG, and identification of damage using biomarkers in blood.^209^ While the latter are available as in vitro assays, other readouts are very different from those used to assess 3D cardiac models. For example, electrical field potentials measured using multielectrode arrays in vitro have provided estimates of QT prolongation^210^ and CV,^211^ but this is only a fraction of the information provided by a 12-lead ECG. Engineering 3D multielectrode arrays in combination with mathematical modeling might result in directly translatable readouts of electrical activity. For assessment of contractility, miniaturized ventricular chambers have been developed that can estimate ejection fraction output,^109^ but these did not show the physiologically expected increase in ejection fraction with isoproterenol. Another approach might be to use the same image-based quantification technology, such as MUSCLEMOTION,^13^ for both in vitro and in vivo measurements, but this would require additional mathematical modeling to be able to translate the outcome to clinically meaningful results.

A critical point to develop and validate mathematical models for translational purposes is to compare clinical data with measurements in personalized cardiac 3D models based preferably on patient-specific cells with their isogenic controls. This could eventually demonstrate which model can capture specific complex disease phenotypes and finally contribute to finding novel therapies.

## Conclusions

3D cardiac tissues clearly exhibit many features of native human myocardium, albeit at early postnatal or juvenile stages of development. There are many biophysical conditions that could still be used in combination to advance these models further and increase their utility in disease modeling, revealing the role of novel genetic variants of unknown significance and setting the stage for developing new treatments that are currently unavailable for many chronic conditions of the heart. Providing better and more accessible models is also an important contribution that could be made by academia to the pharmaceutical industry—an incentive to increase efforts not only to treat (rare) genetic conditions but also those resulting from environmental factors, such as lifestyle or side effects of, for example, cancer therapy. Importantly, interaction between environmental and genetic predisposition might be captured using patient hiPSCs.

## Acknowledgments

Figures were created with BioRender.com.

## Sources of Funding

This study was funded by the Netherlands Organ-on-Chip Initiative, an NWO Gravitation project funded by the Ministry of Education, Culture and Science of the government of the Netherlands (024.003.001); European Union Horizon 2020 Research and Innovation Programme under the Marie Sklodowska Curie Actions (individual fellowship, MSCA-IF 838985 SiGNATURE); Transnational Research Project on Cardiovascular Diseases (JTC2016_FP-40-021); and the Netherlands Organisation for Health Research and Development ZonMW (Meer Kennis met Minder Dieren [MKMD]), project No. 114022504).

## Disclosures

None.
